# *Guilotes*, a new genus of Coelotinae spiders from Guangxi Zhuang Autonomous Region, China (Araneae, Agelenidae)

**DOI:** 10.3897/zookeys.802.29913

**Published:** 2018-12-04

**Authors:** Bing Li, Zhe Zhao, Haifeng Chen, Zhiyan Wu, Chuntian Zhang, Shuqiang Li

**Affiliations:** 1 College of Life Science, Langfang Normal University, Langfang 065000, Hebei Province, China Langfang Normal University Langfang China; 2 College of Life Science, Shenyang Normal University, Shenyang, Liaoning 110034, China Shenyang Normal University Liaoning China; 3 Institute of Zoology, Chinese Academy of Sciences, Beijing 100101, China Institute of Zoology, Chinese Academy of Sciences Beijing China

**Keywords:** Asia, barcodes, new species, taxonomy

## Abstract

A new genus of the subfamily Coelotinae F.O. Pickard-Cambridge, 1893, *Guilotes* Z. Zhao & S. Li, **gen. n.** from China is described, as well as four new species: *G.ludiensis* Z. Zhao & S. Li, **sp. n.** (♂♀, type species), *G.qingshitanensis* Z. Zhao & S. Li, **sp. n.** (♂♀), *G.xingpingensis* Z. Zhao & S. Li, **sp. n.** (♂♀) and *G.yandongensis* Z. Zhao & S. Li, **sp. n.** (♀). The DNA barcodes of all species are documented for future use.

## Introduction

The spider subfamily Coelotinae (Araneae, Agelenidae) comprises 743 valid species belonging to 30 genera (World Spider Catalog 2018; Li and Quan 2017). Coelotinae are recorded only in the Nearctic, Palearctic, and Indo-Malaya regions with 89% of the species distributed in Asia, 7% in Europe, and 4% in North America. New coelotine genera and species had been recently discovered (Chen et al. 2015a, b, 2016a, b; Zhang and Marusik 2016; Zhang et al. 2016a, b; Zhao and Li 2016; Komnenov 2017; Okumura 2017; Quasin et al. 2017; Zhang and Zhao 2017; Zhang et al. 2017; Zhu et al. 2017; Li et al. 2018a, b). Zhao and Li (2017) studied the evolutionary history and biogeography of Coelotinae using molecular data (8 genes, ~ 6.5 kb) on 18 genera and 286 coelotine species. The well resolved phylogeny of coelotine spiders promoted the new taxa erecting and taxonomic revisions (Chen et al. 2016b; Zhao and Li 2016; Li et al. 2018a, b). So far, there are 387 coelotine species (about 52% of the total) from 24 genera reported from China. However, in the karst regions of China, there are still many poorly known species with unusual characters which are not belonging to any known coelotine genera (World Spider Catalog 2018).

In this study, *Guilotes* Z. Zhao & S. Li, gen. n. is proposed to include four new species. The new genus was confirmed by the phylogenetic framework of coelotine spiders (Zhao and Li 2017). The molecular topologies supported *Guilotes* as a monophyletic group in Guangxi *Coelotes* groups. The new species of *Guilotes* are compared with those of the morphological similar genus *Notiocoelotes* Wang, Xu & Li, 2008. All specimens of *Guilotes* were collected from karst regions in southern China.

## Materials and methods

Specimens were examined with a LEICA M205C stereomicroscope. Photographs were captured with an Olympus C7070 wide zoom digital camera (7.1 megapixels) mounted either on an Olympus SZX12 dissecting microscope or on an Olympus BX51 compound microscope. Photos from multiple focal ranges were combined using Helicon Focus (Version 3.10) photo stacking software. Female epigyne and male palp were dissected form the body to be proper examined. The epigyne was removed and treated in a warmed 10% potassium hydroxide (KOH) solution before study. Measurements were obtained with a LEICA M205C stereomicroscope and are given in millimeters. Eye diameters were measured as the maximum diameter from either dorsal or frontal views. Leg measurements are given as: total length (femur, patella + tibia, metatarsus, tarsus). Images of the male left palp are presented. Terminology of the structures follows Wang (2002), Chen et al. (2015b) and Zhang et al. (2016b).

References to figures in the cited papers are listed in lowercase (figure or figs); figures from this paper are noted with an initial capital (Figure or Figs). Abbreviations used in the text and figures:

**A** epigynal atrium;

**ALE** anterior lateral eye;

**ALE–PLE** distance between ALE and PLE;

**AME** anterior median eye;

**AME–ALE** distance between AME and ALE;

**AME–AME** distance between AME and AME;

**AME – PME** distance between AME and PME;

**C** conductor;

**CD** copulatory duct;

**CF** cymbial furrow;

**CO** copulatory opening;

**E** embolus;

**EB** embolic base;

**ET** epigynal tooth;

**FD** fertilization duct;

**LC** lamella of conductor;

**LTA** lateral tibial apophysis;

**MA** median apophysis;

**OC** outgrowth of conductor;

**PA** patellar apophysis;

**PLE** posterior lateral eye;

**PME** posterior median eye;

**PME–PLE** distance between PME and PLE;

**PME–PME** distance between PME and PME;

**RTA** retrolateral tibial apophysis;

**S** spermatheca;

**SB** spermathecal base;

**SH** spermathecal head;

**ST** subtegulum;

**T** tegulum;

**TS** tegular sclerite.

DNA barcodes were also obtained for the species delimitation and matching. A partial fragment of the mitochondrial cytochrome oxidase subunit I (*CO1*) gene was amplified and sequenced for all species using the primers LCO1490-oono (5’-CWACAAAYCATARRGATATTGG-3’) and C1-N-2776 (5’-GGATAATCAGAATANCGNCGAGG-3’). For additional information on extraction, amplification and sequencing procedures, see Zhao and Li (2017). All sequences were analyzed using BLAST and are deposited in GenBank. The accession numbers are provided in Table 1.

**Table 1.** Voucher specimen information.

*Guilotes* species	Voucher code	GenBank accession number	Sequence length	Collection localities
*G.ludiensis* sp. n.	IZCAS-Ar34051(zz391)	KY778823	1194bp	Guilin City, Guangxi, China
*G.qingshitanensis* sp. n.	IZCAS-Ar34059(zz892)	KY778825	1194bp	Guilin City, Guangxi, China
*G.xingpingensis* sp. n.	IZCAS-Ar34067(zz890)	KY778824	1194bp	Guilin City, Guangxi, China
*G.yandongensis* sp. n.	IZCAS-Ar34075(zz392)	KY778813	1194bp	Baise City, Guangxi, China

All specimens (including molecular vouchers) are deposited in the Institute of Zoology, Chinese Academy of Sciences (**IZCAS**) Beijing, China.

## Taxonomy

### Family Agelenidae C.L. Koch, 1837

#### Subfamily Coelotinae F.O. Pickard-Cambridge, 1893

##### Genus Guilotes

Taxon classificationAnimaliaAraneaeAgelenidae

Z. Zhao & S. Li gen. n.

http://zoobank.org/024F72C8-3B81-4F0A-96C6-B25424ADB896

Figs 1 , 2 , 3 , 4 , 5 , 6 , 7 , 8

###### Type species.

*Guilotesludiensis* Z. Zhao & S. Li, sp. n.

###### Etymology.

The generic name is derived from the pinyin word “Gui”, referring to the Guangxi Zhuang Autonomous Region (Gui is a short name for Guangxi) where the genus is distributed, and “-*lotes*” as part of *Notiocoelotes*, which is similar to the new genus. The gender is masculine.

###### Diagnosis.

**Figure 1.**
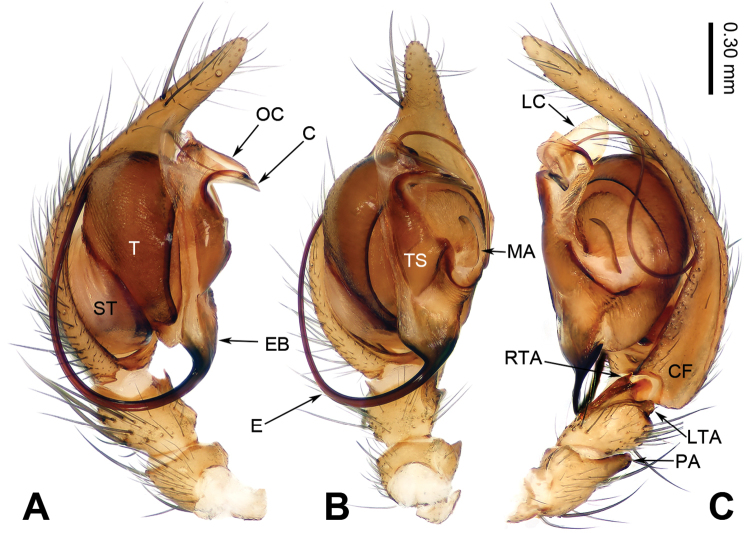
Left male palp of *Guilotesludiensis* sp. n., holotype. **A** Prolateral view **B** Ventral view **C** Retrolateral view.

**Figure 2.**
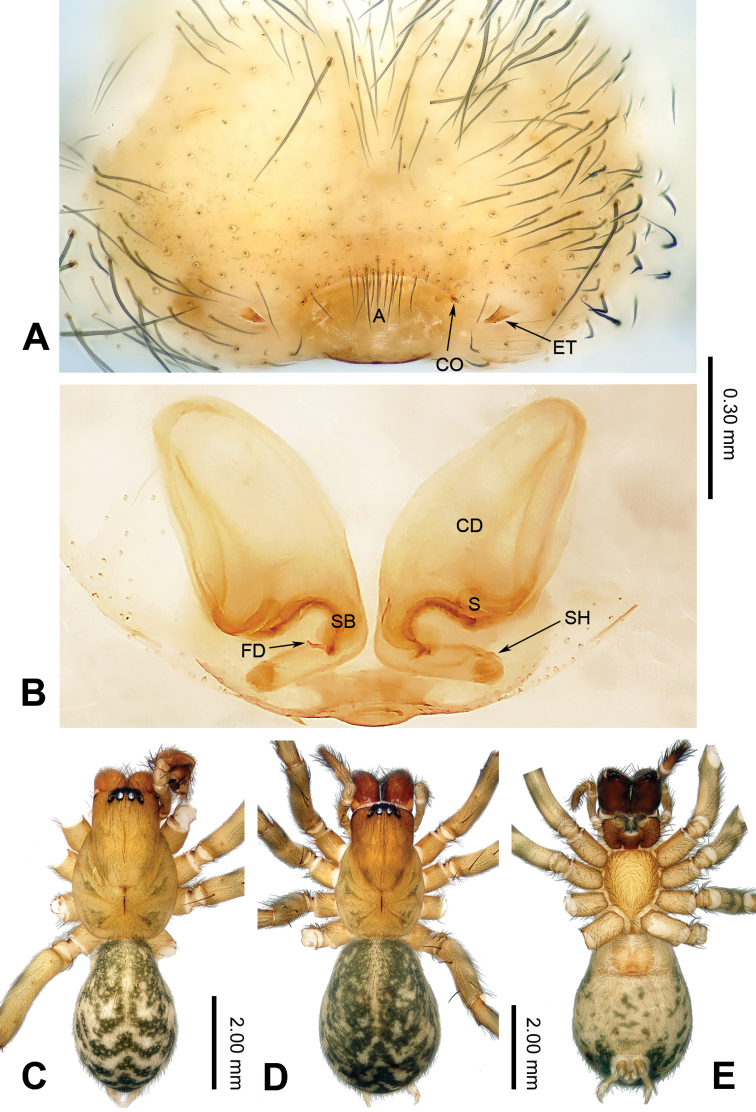
Epigyne and habitus of *Guilotesludiensis* sp. n. **A** Epigyne, ventral **B** Vulva, dorsal **C** Male habitus, dorsal **D** Female habitus, dorsal **E** Female habitus, ventral. Scale bar equal for **D** and **E**.

**Figure 3.**
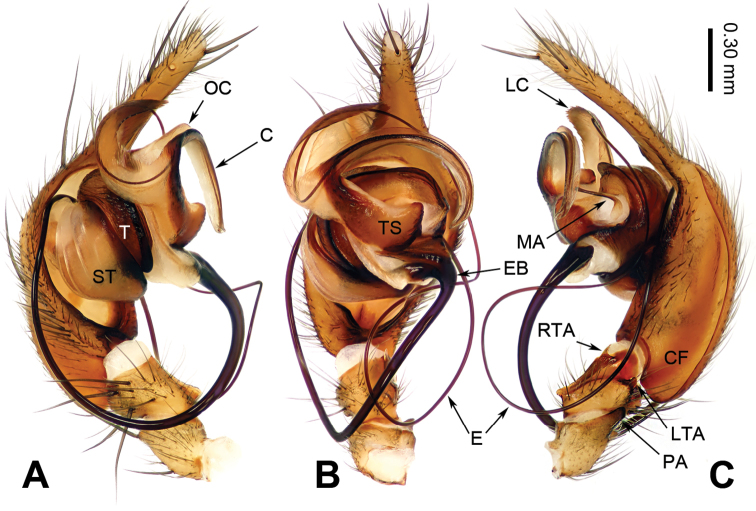
Left male palp of *Guilotesqingshitanensis* sp. n., holotype. **A** Prolateral view **B** Ventral view **C** Retrolateral view.

**Figure 4.**
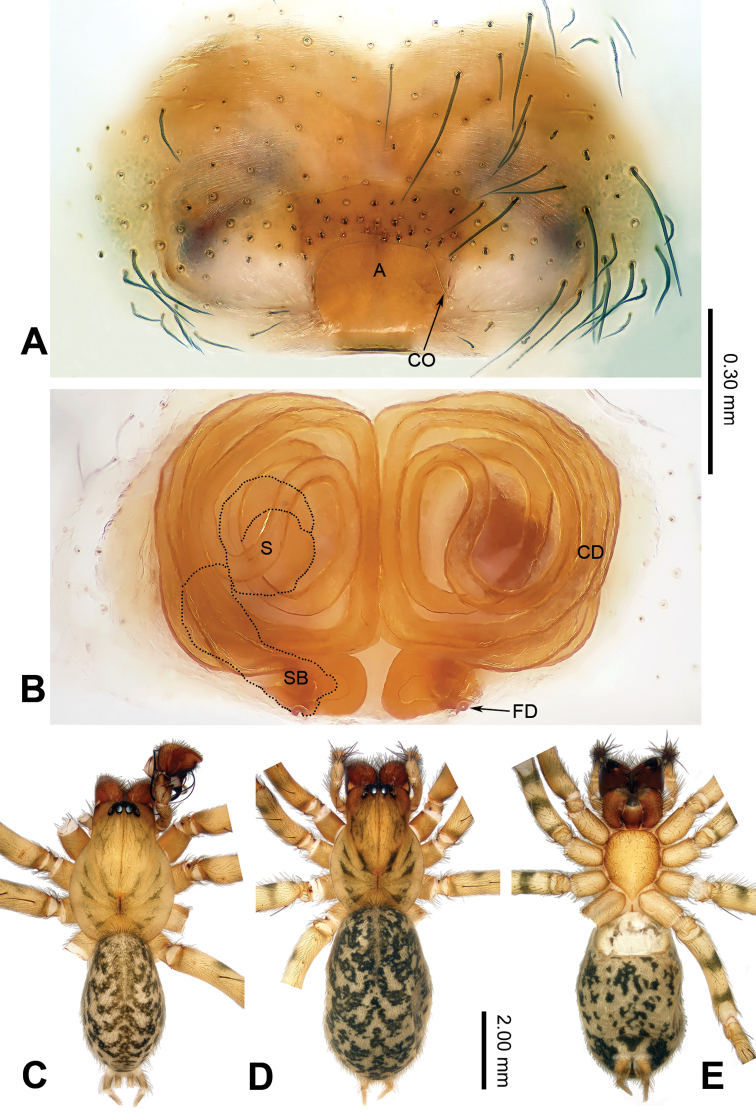
Epigyne and habitus of *Guilotesqingshitanensis* sp. n. **A** Epigyne, ventral **B** Vulva, dorsal **C** Male habitus, dorsal **D** Female habitus, dorsal **E** Female habitus, ventral. Scale bars equal for **C–E**.

**Figure 5.**
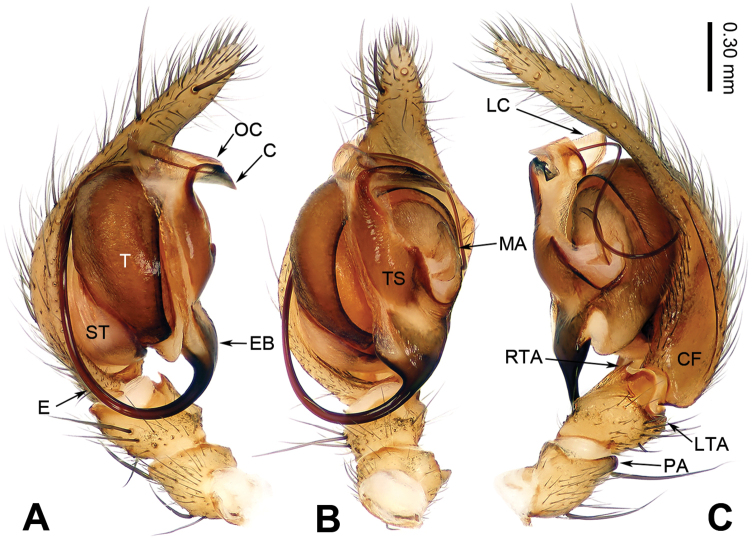
Left male palp of *Guilotesxingpingensis* sp. n., holotype. **A** Prolateral view **B** Ventral view **C** Retrolateral view.

**Figure 6.**
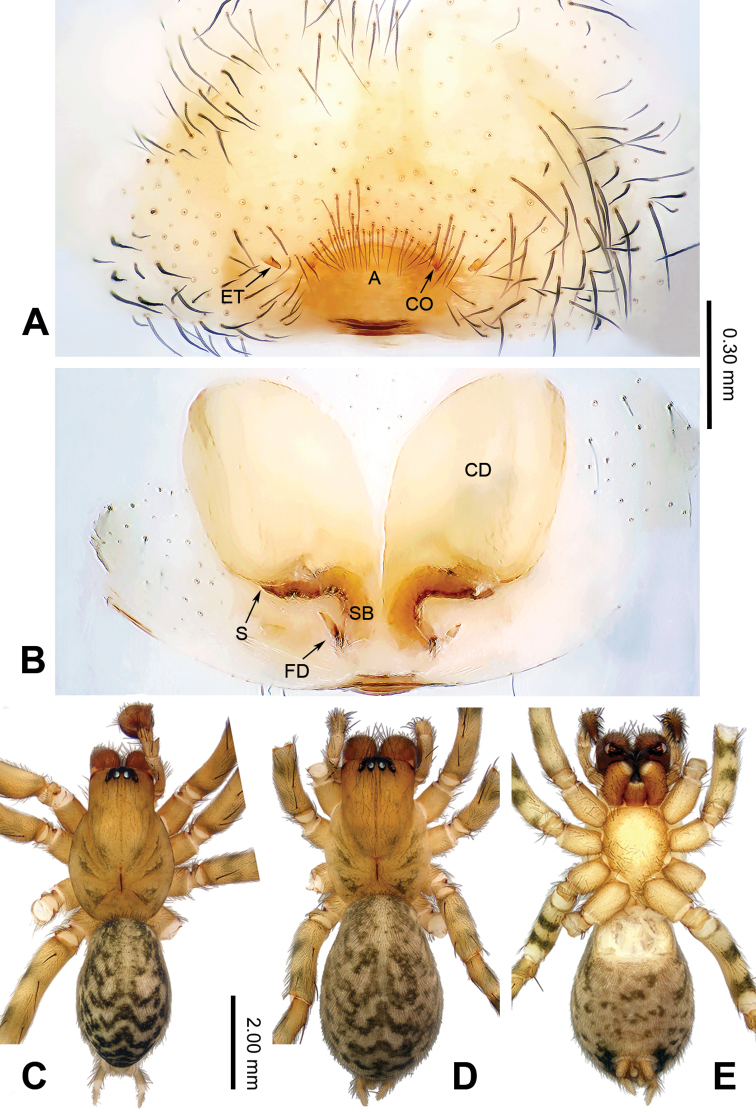
Epigyne and habitus of *Guilotesxingpingensis* sp. n. **A** Epigyne, ventral **B** Vulva, dorsal **C** Male habitus, dorsal **D** Female habitus, dorsal **E** Female habitus, ventral. Scale bars equal for **C–E**.

**Figure 7.**
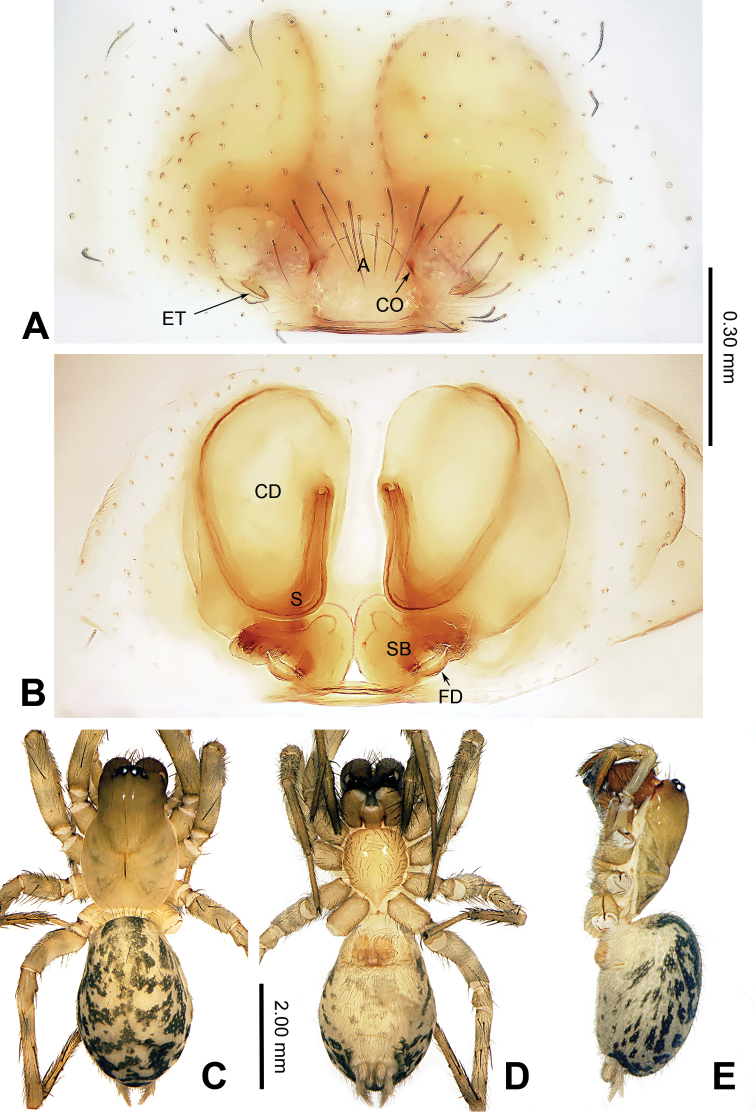
Epigyne and habitus of *Guilotesyandongensis* sp. n. **A** Epigyne, ventral **B** Vulva, dorsal **C** Female habitus, dorsal **D** Female habitus, ventral **E** Female habitus, lateral. Scale bars equal for **C–E**.

The males of the genus *Guilotes* are similar to those of the genus *Notiocoelotes* by cymbial furrow long (Figs 1C, 3C, 5C) and embolus long and filiform (Figs 1B, 3B, 5B), but can be distinguished by the chelicerae with 5–6 promarginal and five retromarginal teeth; the presence of a patellar apophysis (Figure 1C) and conductor short not reaching the embolus base (Figure 1B). The females of the genus *Guilotes* are similar to those of the genus *Notiocoelotes* by atrium oval and posterior (Figs 2A, 4A, 6A, 7A), hoods absent, copulatory ducts large, but can be distinguished by the chelicerae with 4–6 promarginal and 5–6 retromarginal teeth, the absence of a tongue-shaped atrial scape (Figure 2A), and the presence of two epigynal teeth (Figure 2A, absent in *G.qingshitanensis* sp. n. (Figure 4A)).

###### Description.

Medium sized spiders, with total length 4.17–8.66. Carapace yellowish brown; cephalic area, labium, endites and sternum brown; chelicerae dark brown (Figs 2C–E, 4C–E, 6C–E, 7C–E). Abdomen gray with dark gray chevron stripes (Figs 2C–D, 4C–D, 6C–D, 7C). Spinnerets yellowish brown (Figs 2C–E, 4C–E, 6C–E, 7C–E). Legs yellowish brown with dark rings (Figs 2C–E, 4C–E, 6C–E, 7C–E). Chelicerae usually with 4–6 promarginal and 5–6 retromarginal teeth. Anterior lateral spinnerets larger than posterior median spinnerets but smaller than posterior lateral spinnerets; colulus slide-shaped; distal article of the spinneret longer than coxa. Leg formula 4123. Palp: patellar apophysis finger-shaped, RTA large and LTA small (Figs 1C, 3C, 5C). Cymbial furrow equal to (Figs 1C, 5C) or longer (Figure 3C) than 1/2 length of cymbium. Embolus long and filiform, beginning at 4 to 5 o’clock position (Figs 1B, 3B, 5B). Conductor short and broad with an outgrowth (Figs 1A, 3A, 5A) and a dorsal lamella (LC = lamella of conductor; Figs 1C, 3C, 5C). Median apophysis broad (Figs 1C, 5C) or thin, sharp and elongated (Figure 3C). Epigyne: with two teeth (Figs 2A, 6A, 7A, absent in *G.qingshitanensis* sp. n. (Figure 4A)). Atrium oval and posterior (Figs 2A, 4A, 6A, 7A). Hoods absent. Copulatory openings centrally located (on corners of the atrium; Figs 2A, 4A, 6A, 7A). Copulatory duct expanded and sac-like (Figs 2B, 6B, 7B) or elongate and convoluted (Figure 4B). Spermathecae long, spermathecae heads long, stretched to the back (Figs 2B, 4B, 6B, 7B).

###### Natural history.

All species of this genus were collected from moist caves with soluble rock.

###### Comments.

The new genus was supported as monophyletic within the Guangxi *Coelotes* group (Zhao and Li 2017: 993, figure 3 - see ZZ391, ZZ392, ZZ890, and ZZ892). Males and females of each species were collected from the same caves and double-checked using DNA barcoding.

The divergence time analyses showed the split of *Guilotes* gen. n. and other close related clades early than 30 million years ago (Zhao and Li 2017: figure 3, S8). *Guilotes* and *Notiocoelotes* are very similar in morphology and present similar distribution areas (Wang et al. 2008, Liu et al. 2010, Zhang et al. 2016b); thus, it is meaningful to distinguish between them.

###### Distribution.

**Figure 8.**
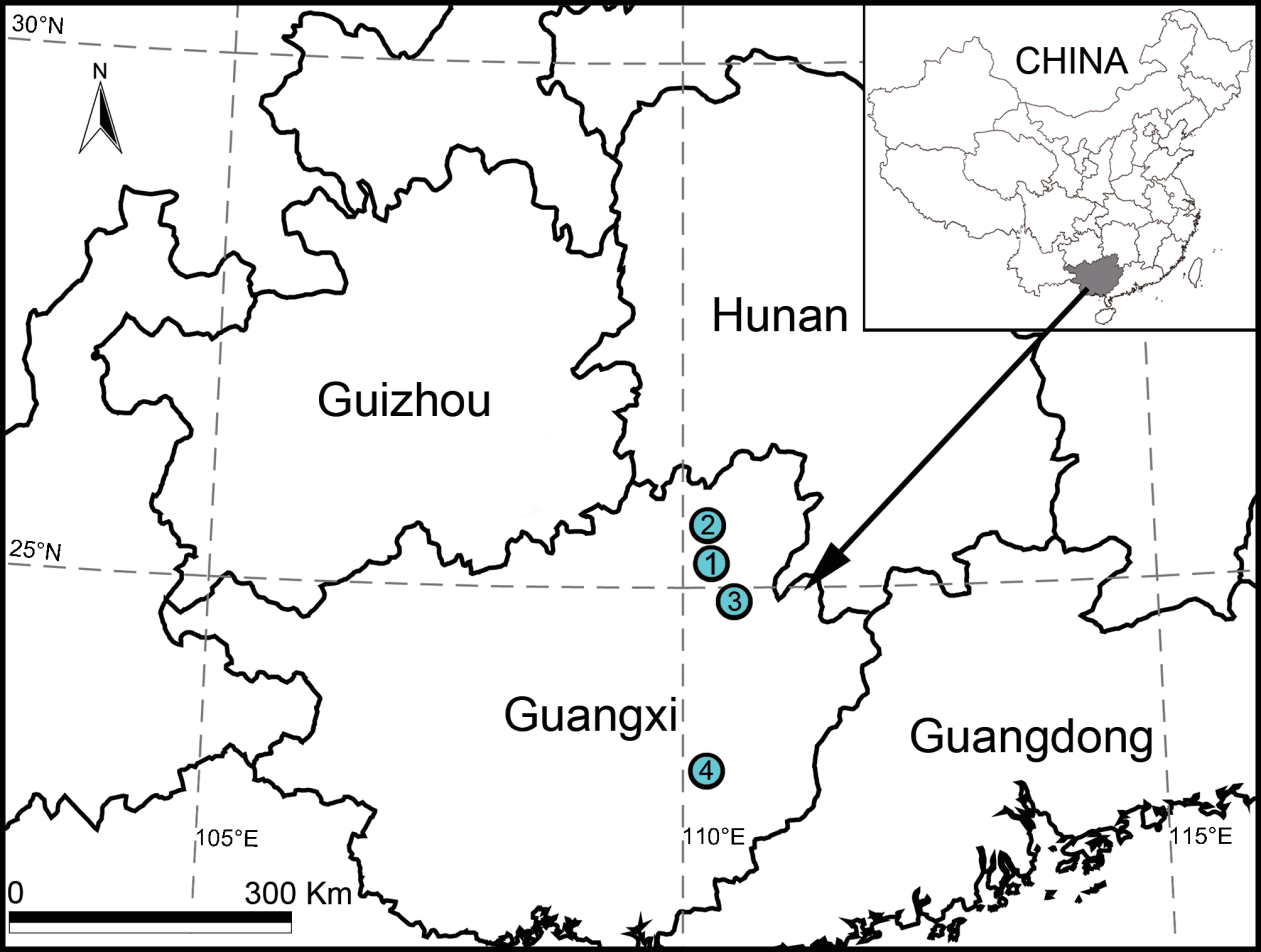
Localities of *Guilotes* species in China. **1***G.ludiensis* sp. n. **2***G.qingshitanensis* sp. n. **3***G.xingpingensis* sp. n. **4***G.yandongensis* sp. n.

Guangxi Zhuang Autonomous Region, China (Figure 8).

##### Guilotes ludiensis

Taxon classificationAnimaliaAraneaeAgelenidae

Z. Zhao & S. Li sp. n.

http://zoobank.org/BA5B15BE-8B5C-4D02-9FF3-CD43C6073703

Figs 1 , 2 , 8

###### Type material.

**Holotype** ♂ (IZCAS-Ar34050): China: Guangxi Zhuang Autonomous Region: Guilin City: Ludi Cave, 25°18.551'N, 110°15.822'E, elevation: 200 m, 6.XII.2015, Z. Chen and X. Zhang leg. **Paratypes**: 1♀ (IZCAS-Ar34051, zz391, KY778823), same cave as holotype, 25°18.505'N, 110°15.793'E, elevation: 200 m, 9.XII.2012, Z. Chen and Z. Zhao leg.; 1♂3♀♀ (IZCAS-Ar34052–Ar34055), same data as holotype; 2♀♀ (IZCAS-Ar34056, Ar34057), same cave as the holotype, 25°18.237'N, 110°16.218'E, elevation: 150 ± 3 m, 5–6.I.2018, Z. Chen leg.

###### Etymology.

The specific name is an adjective and refers to the type locality, Ludi Cave.

###### Diagnosis.

Males of *Guilotesludiensis* sp. n. can be distinguished from *G.xingpingensis* sp. n. with the long patellar apophysis, wide lateral tibial apophysis (Figs 1C, 5C) and narrow conductor (Figs 1A, 5A) with swollen OC and large LC (Figure 1C). Differ from *G.qingshitanensis* sp. n. by the straight and short conductor with a LC and embolus beginning at 5 o’clock position (Figure 1B). Females can be distinguished from *G.xingpingensis* sp. n. by the epigynal teeth located in the middle of the atrial lateral margins (Figure 2A), copulatory ducts concave laterally and narrow fertilization ducts (Figure 2B). Differ from *G.qingshitanensis* sp. n. by the presence of epigynal teeth (Figure 2A) and sac-like of copulatory ducts (Figs 2B, 4B). Differ from *G.yandongensis* sp. n. by locations of epigynal teeth near the atrial lateral margins, atrium twice wider than long (Figure 2A), copulatory ducts ending horizontally and narrow fertilization ducts (Figure 2B).

###### Description.

**Male** (holotype, IZCAS-Ar34050): Total length 5.94. Carapace 3.56 long, 2.77 wide. Abdomen 2.38 long, 1.78 wide. Eye diameters and interdistances: AME: 0.08, ALE: 0.16, PME: 0.15, PLE: 0.15; AME–AME: 0.04, AME–ALE: 0.03, AME–PME: 0.10, ALE–PLE: 0.02, PME–PME: 0.05, PME–PLE: 0.05. Leg measurements: I: 11.22 (3.96, 3.26, 2.60, 1.40); II: 10.51 (3.61, 3.36, 2.18, 1.36); III: 10.01 (3.39, 3.05, 2.38, 1.19); IV: 12.41 (4.17, 3.68, 3.17, 1.39). Chelicerae with five promarginal and five retromarginal teeth. Palp: patellar apophysis finger-shaped, its length shorter than width of patella (Figure 1C); RTA narrow, pointed tip (Figure 1C); LTA short, approximately 1/2 length of RTA (Figure 1C); cymbial furrow long, subequal to 2/3 length of cymbium (Figure 1C); embolus filiform, beginning at 5 o’clock position (Figure 1B); conductor short, horizontally directed (Figure 1A–B); OC located at the base of the conductor (Figure 1A) and LC located behind the outgrowth (Figure 1C); median apophysis small, spoon-like (Figure 1B–C).

**Female** (one of the paratypes, IZCAS-Ar34053): Total length 5.54. Carapace 2.57 long, 1.44 wide. Abdomen 2.97 long, 2.08 wide. Eye diameters and interdistances: AME: 0.05, ALE: 0.12, PME: 0.10, PLE: 0.11; AME–AME: 0.06, AME–ALE: 0.03, AME–PME: 0.08, ALE–PLE: 0.05, PME–PME: 0.08, PME–PLE: 0.07. Leg measurements: I: 7.08 (2.59, 2.21, 1.42, 0.86); II: 6.13 (2.06, 1.80, 1.46, 0.81); III: 5.52 (2.01, 1.61, 1.21, 0.69); IV: 7.66 (2.79, 2.41, 1.61, 0.85). Chelicerae with six promarginal and five or six retromarginal teeth. Epigyne: teeth short, less than 1/2 atrial length, located near the atrial lateral margins (Figure 2A); atrium small, occupying 1/7 epigynal plate (Figure 2A); copulatory ducts broad, occupying 3/4 epigynal plate (Figure 2B); spermathecae cylindrical, elongated and posterior, stay away from each other (Figure 2B); spermathecal heads long, stretched to the back (Figure 2B).

###### Variation.

Total length: males 5.94–6.53 (n = 2); females 5.54–7.13 (n = 6).

###### Distribution.

Males and females of this species were collected from Ludi Cave, Guilin City, Guangxi Zhuang Autonomous Region, China (Figure 8).

##### Guilotes qingshitanensis

Taxon classificationAnimaliaAraneaeAgelenidae

Z. Zhao & S. Li sp. n.

http://zoobank.org/55071426-8B9F-4386-9A9E-9EA29B53AF78

Figs 3 , 4 , 8

###### Type material.

**Holotype** ♂ (IZCAS-Ar34058): China: Guangxi Zhuang Autonomous Region: Guilin City: Lingchuan County, Qingshitan Town, Yanbei Village, Yanbei Cave, 25°30.622'N, 110°14.969'E, elevation: 173 m, 7.XII.2015, X. Zhang and Z. Chen leg. **Paratypes**: 1♀ (IZCAS-Ar34059, zz892, KY778825), same cave as holotype, 25°31.137'N, 110°14.908'E, elevation: 173 m, 21.XII.2013, H. Zhao leg.; 1♂2♀♀ (IZCAS-Ar34060–Ar34062), same data as holotype; 2♂♂2♀♀ (IZCAS-Ar34063–Ar34066), same cave as holotype, 25°31.607'N, 110°14.967'E, elevation: 201 ± 4 m, 3.I.2018, Z. Chen leg.

###### Etymology.

The specific name is an adjective and refers to the type locality, Qingshitan Town.

###### Diagnosis.

Males of *Guilotesqingshitanensis* sp. n. differ from *G.ludiensis* sp. n. and *G.xingpingensis* sp. n. by long and bent conductor and OC (Figure 3A–B), LC with saw-shaped margin (Figure 3C), embolus beginning at 4 o’clock position (Figure 3B), small median apophysis with needle-shaped top and spoon-shaped end (Figure 3B–C). Females differ from *G.ludiensis* sp. n., *G.xingpingensis* sp. n. and *G.yandongensis* sp. n. by the absence of epigynal teeth (Figure 4A) and spiral copulatory ducts (Figure 4B).

###### Description.

**Male** (holotype, IZCAS-Ar34058): Total length 6.92. Carapace 3.53 long, 2.51 wide. Abdomen 3.39 long, 2.01 wide. Eye diameters and interdistances: AME: 0.08, ALE: 0.15, PME: 0.14, PLE: 0.16; AME–AME: 0.06, AME–ALE: 0.05, AME–PME: 0.08, ALE–PLE: 0.04, PME–PME: 0.10, PME–PLE: 0.10. Leg measurements: I: 12.64 (4.96, 3.65, 2.25, 1.78); II: 11.08 (4.22, 3.21, 2.24, 1.41); III: 9.69 (3.85, 2.56, 2.44, 0.84); IV: 14.02 (5.02, 3.97, 3.43, 1.60). Chelicerae with six promarginal and five retromarginal teeth. Palp: patellar apophysis finger-shaped, its length subequal to width of patella (Figure 3C); RTA narrow, keel-shaped apex (Figure 3C); LTA short, approximately 1/5 length of RTA (Figure 3C); cymbial furrow long, subequal to 1/2 length of cymbium (Figure 3C); embolus filiform, beginning at 4 o’clock position (Figure 3B–C); conductor long and bent (Figure 3A); outgrowth of the conductor beginning at the base of conductor, with similar shape as conductor (Figure 3A); lamella of the conductor broad, with saw-shaped margin (Figure 3C); median apophysis small, its end spoon-shaped while its top needle-shaped (Figure 3C).

**Female** (one of the paratypes, IZCAS-Ar34061): Total length 7.82. Carapace 3.53 long, 2.35 wide. Abdomen 4.29 long, 3.01 wide. Eye diameters and interdistances: AME: 0.09, ALE: 0.16, PME: 0.16, PLE: 0.15; AME–AME: 0.08, AME–ALE: 0.05, AME–PME: 0.08, ALE–PLE: 0.04, PME–PME: 0.10, PME–PLE: 0.09. Leg measurements: I: 9.74 (2.69, 3.27, 2.24, 1.54); II: 8.71 (2.82, 2.69, 1.92, 1.28); III: 8.14 (2.69, 2.37, 1.99, 1.09); IV: 10.97 (3.08, 3.53, 3.01, 1.35). Chelicerae with five or six promarginal and five retromarginal teeth. Epigyne: teeth absent (Figure 4A); atrium small, occupying 1/7 epigynal plate (Figure 4A); copulatory ducts long and spiral (Figure 4B); spermathecae long and longitudinally lengthening (Figure 4B); spermathecal heads and stalk covered by the copulatory ducts in dorsal view (Figure 4B); spermathecal bases horizontally extended (Figure 4B).

###### Variation.

Total length: males 6.92–7.69 (n = 4); females 5.54–7.13 (n = 5).

###### Distribution.

Males and females of this species were collected from Yanbei Cave, Guilin City, Guangxi Zhuang Autonomous Region, China (Figure 8).

##### Guilotes xingpingensis

Taxon classificationAnimaliaAraneaeAgelenidae

Z. Zhao & S. Li sp. n.

http://zoobank.org/181B0155-FC02-4D9C-B169-25CD54DDB6A4

Figs 5 , 6 , 8

###### Type material.

**Holotype** ♂ (IZCAS-Ar34067, zz890, KY778824): China: Guangxi Zhuang Autonomous Region: Guilin City: Yangshuo County, Xingping Town, Luotian Village, Luotian Cave, 24°56.731'N, 110°31.459'E, elevation: 217 m, 17.XII.2013, H. Zhao leg. **Paratypes**: 3♂♂4♀♀ (IZCAS-Ar34068–Ar34074), same cave as holotype, elevation: 241 m, 8.XII.2015, X. Zhang and Z. Chen leg.

###### Etymology.

The specific name is an adjective and refers to the type locality, Xingping Town.

###### Diagnosis.

Males of *Guilotesxingpingensis* sp. n. differ from *G.ludiensis* sp. n. by the patellar apophysis short, lateral tibial apophysis narrow, conductor wide with flat outgrowth and small lamella (Figure 5C). Females differ from *G.ludiensis* sp. n. by the sail-shaped copulatory ducts and fertilization ducts wide and long (Figure 6B).

###### Description.

**Male** (holotype, IZCAS-Ar34067): Total length 5.03. Carapace 2.65 long, 1.75 wide. Abdomen 2.38 long, 1.56 wide. Eye diameters and interdistances: AME: 0.06, ALE: 0.13, PME: 0.12, PLE: 0.13; AME–AME: 0.07, AME–ALE: 0.06, AME–PME: 0.08, ALE–PLE: 0.02, PME–PME: 0.10, PME–PLE: 0.11. Leg measurements: I: 9.68 (3.28, 2.97, 2.09, 1.34); II: 7.84 (2.56, 2.31, 1.88, 1.09); III: 7.32 (2.56, 1.94, 1.88, 0.94); IV: 10.20 (3.44, 2.97, 2.66, 1.13). Chelicerae with six promarginal and five retromarginal teeth. Palp: patellar apophysis short, its length approximately half length of patella, finger-shaped (Figure 5C); RTA narrow, pointed tip (Figure 5C); LTA short, approximately 1/4 length of RTA (Figure 5C); cymbial furrow long, subequal to 1/2 length of cymbium (Figure 5C); embolus filiform, beginning at 5 o’clock position (Figure 5A, B); conductor short, horizontally directed (Figure 5A); base of conductor with one outgrowth (Figure 5A); lamella of the conductor located behind the outgrowth (Figure 5C); median apophysis small, spoon-shaped (Figure 5B–C).

**Female** (one of the paratype, IZCAS-Ar34071): Total length 6.99. Carapace 3.21 long, 2.24 wide. Abdomen 3.78 long, 2.44 wide. Eye diameters and interdistances: AME: 0.06, ALE: 0.12, PME: 0.11, PLE: 0.12; AME–AME: 0.07, AME–ALE: 0.04, AME–PME: 0.08, ALE–PLE: 0.04, PME–PME: 0.09, PME–PLE: 0.09. Leg measurements: I: 9.55 (3.72, 2.56, 2.12, 1.15); II: 8.49 (3.72, 2.28, 1.53, 0.96); III: 7.89 (3.09, 2.12, 1.73, 0.95); IV: 10.71 (4.11, 3.14, 2.05, 1.41). Chelicerae with five promarginal and five retromarginal teeth. Epigyne: teeth short, about 1/4 atrial length, located near the atrial lateral margins (Figure 6A); atrium small, occupying 1/8 epigynal plate (Figure 6A); copulatory ducts broad, occupying 3/4 epigynal plate (Figure 6B); spermathecae bean-shaped with complex lumen and posterior, stay away from each other, and below the copulatory ducts (Figure 6B); spermathecal heads long, stretched to the back (Figure 6B).

###### Variation.

Total length: males 4.17–6.41 (n = 4); females 4.40–6.99 (n = 4).

###### Distribution.

Males and females of this species were collected from Luotian Cave, Guilin City, Guangxi Zhuang Autonomous Region, China (Figure 8).

##### Guilotes yandongensis

Taxon classificationAnimaliaAraneaeAgelenidae

Z. Zhao & S. Li sp. n.

http://zoobank.org/315FCE4E-69D1-4138-994C-8953E4A8F0E8

Figs 7 , 8

###### Type material.

**Holotype** ♀ (IZCAS-Ar34075, zz392, KY778813): China: Guangxi Zhuang Autonomous Region: Baise City: Debao County, Yandong Town, Xingwang Village, Podi Cave, 23°14.268'N, 110°14.597'E, elevation: 632 m, 9.XII.2012, Z. Zhao and Z. Chen leg. **Paratypes**: 2♀♀ (IZCAS-Ar34076, Ar34077), same cave as holotype, 11.XII.2015, X. Zhang and Z. Chen leg.

###### Etymology.

The specific name is an adjective and refers to the type locality, Podi Cave.

###### Diagnosis.

Females of *Guilotesyandongensis* sp. n. can be differ from *G.qingshitanensis* sp. n. by having the epigynal teeth (Figure 7A) and wide fertilization duct (Figure 7B); they differ from *G.ludiensis* sp. n. and *G.xingpingensis* sp. n. by the copulatory ducts with folded lateral margin (Figure 7B).

###### Description.

**Female** (holotype, IZCAS-Ar34075): Total length 5.77. Carapace 2.51 long, 1.61 wide. Abdomen 3.26 long, 2.05 wide. Eye diameters and interdistances: AME: 0.08, ALE: 0.12, PME: 0.12, PLE: 0.12; AME–AME: 0.04, AME–ALE: 0.04, AME–PME: 0.08, ALE–PLE: 0.05, PME–PME: 0.09, PME–PLE: 0.07. Leg measurements: I: 7.49 (2.81, 2.34, 1.25, 1.09); II: 7.11 (2.67, 2.03, 1.38, 1.03); III: 6.32 (2.19, 1.88, 1.47, 0.78); IV: 8.65 (2.81, 2.59, 2.19, 1.06). Chelicerae with four promarginal and five retromarginal teeth. Epigyne: teeth short, subequal to 1/3 atrial length, located far from the atrial lateral margins (Figure 7A); atrium small, occupying less than 1/8 epigynal plate (Figure 7A); copulatory ducts broad, occupying 3/4 epigynal plate, with folded lateral margin (Figure 7B); spermathecae long (Figure 7B); spermathecal heads long, stretched to the back (Figure 7B); spermathecal stalks long, lengthening along the margin of copulatory ducts (Figure 7B).

###### Variation.

Total length: females 5.77–8.85 (n=3).

###### Distribution.

All specimens of this species were collected from Podi Cave, Baise City, Guangxi Zhuang Autonomous Region, China (Figure 8).

## Supplementary Material

XML Treatment for Guilotes

XML Treatment for Guilotes ludiensis

XML Treatment for Guilotes qingshitanensis

XML Treatment for Guilotes xingpingensis

XML Treatment for Guilotes yandongensis

## References

[B1] Chen L Li S Zhao Z (2015a) A new genus of Coelotinae (Araneae, Agelenidae) from southern China. ZooKeys 541: 41–56. 10.3897/zookeys.541.6678 PMC4714376 26798279

[B2] Chen L Li S Zhao Z (2015b) Five new *Platocoelotes* species (Araneae, Agelenidae) from caves in southern China. ZooKeys 512: 1–18. 10.3897/zookeys.512.9989 PMC4523751 26257557

[B3] Chen L Zhao Z Li S (2016a) Six new species of the spider genus *Spiricoelotes* species (Araneae, Agelenidae) from caves in Jiangxi, China. ZooKeys 561: 1–19. 10.3897/zookeys.561.6965 PMC4768362 27006612

[B4] Chen L Zhao Z Li S (2016b) *Sinocoelotes* gen. n., a new genus of the subfamily Coelotinae (Araneae, Agelenidae) from Southeast Asia. ZooKeys 614: 51–86. 10.3897/zookeys.614.8663 PMC5027656 27667932

[B5] Koch CL (1837) Übersicht des Arachnidensystems. Nürnberg, Heft 1: 1–39. 10.5962/bhl.title.39561

[B6] Komnenov M (2017) New data on spider fauna (Araneae) of Shar Mountain, north-western Macedonia. Proceedings of the 5^th^ Congress of the Ecologists of Macedonia, with international participation (Ohrid, 19–22 October 2016). Special issues of the Macedonian Ecological Society 13: 44–61.

[B7] Li B Zhao Z Zhang C Li S (2018a) *Sinodraconarius* gen. n., a new genus of Coelotinae spiders from Southwest China (Araneae, Agelenidae). Zookeys 770: 117–135. 10.3897/zookeys.770.22470 PMC6041365 30002592

[B8] Li B Zhao Z Zhang C Li S (2018b) *Nuconarius* gen. n. and *Hengconarius* gen. n., two new genera of Coelotinae (Araneae, Agelenidae) spiders from southwest China. Zootaxa 4457(2): 237–262. 10.11646/zootaxa.4457.2.2 30314168

[B9] Li S Quan R (2017) Taxonomy is the cornerstone of biodiversity conservation – SEABRI reports on biological surveys in Southeast Asia. Zoological Research 38(5): 213–214. 10.24272/j.issn.2095-8137.2017.061 PMC5717423 29181897

[B10] Liu J Li S Pham DS (2010) The coelotine spiders from three national parks in northern Vietnam. Zootaxa 2377: 1–93. 10.11646/zootaxa.5537.2.4 39646341

[B11] Okumura KI (2017) *Dichodactylus* gen. nov. (Araneae: Agelenidae: Coelotinae) from Japan. Species Diversity 22: 29–36. 10.12782/sd.22_29

[B12] Pickard-Cambridge FO (1893) Handbook to the study of British spiders (Drassidae and Agalenidae). British Naturalist 3: 117–170.

[B13] Quasin S Siliwal M Uniyal VP (2017) First report of the genus *Draconarius* Ovtchinnikov, 1999 (Araneae: Agelenidae: Coelotinae) with description of a new species from India. European Journal of Zoological Research 5(1): 19–22.

[B14] Wang X (2002) A generic-level revision of the spider subfamily Coelotinae (Araneae, Amaurobiidae). Bulletin of the American Museum of Natural History, 269: 1–150. 10.1206/0003-0090(2002)269%3C0001:AGLROT%3E2.0.CO;2

[B15] Wang X Xu X Li S (2008) *Notiocoelotes*, a new genus of the spider subfamily Coelotinae from southeast Asia (Araneae, Amaurobiidae). Zootaxa 1853: 1–17.

[B16] World Spider Catalog (2018) Natural History Museum Bern. Version 19.0. http://wsc.nmbe.ch [Accessed 26 June 2018]

[B17] Zhang X Marusik YM (2016) A survey of *Pireneitega* from Tajikistan (Agelenidae, Coelotinae). ZooKeys 635: 89–107. 10.3897/zookeys.635.10487 PMC5126511 27917059

[B18] Zhang X Zhao Z (2017) A new species of *Longicoelotes* (Araneae, Agelenidae) from China, with the first description of the male of *L.kulianganus* (Chamberlin, 1924). ZooKeys 686: 137–147. 10.3897/zookeys.686.11711 PMC5672569 29358894

[B19] Zhang X Zhao Z Zheng G Li S (2016a) Nine new species of the spider genus *Pireneitega* Kishida, 1955 (Agelenidae, Coelotinae) from Xinjiang, China. ZooKeys 601: 49–74. 10.3897/zookeys.601.7893 PMC4978079 27551187

[B20] Zhang X Zhao Z Zheng G Li S (2016b) A further study of the spider genus *Notiocoelotes* (Araneae, Agelenidae) from Hainan Island, China. ZooKeys 601: 75–87. 10.3897/zookeys.601.7698 PMC4978080 27551188

[B21] Zhang X Zhao Z Zheng G Li S (2017) A survey of five *Pireneitega* species (Agelenidae, Coelotinae) from China. ZooKeys 663: 45–64. 10.3897/zookeys.663.11356 PMC5523174 28769617

[B22] Zhao Z Li S (2016) *Papiliocoelotes* gen. n., a new genus of Coelotinae (Araneae, Agelenidae) spiders from the Wuling Mountains, China. ZooKeys 585: 33–50. 10.3897/zookeys.585.8007 PMC4857037 27199603

[B23] Zhao Z Li S (2017) Extinction vs. rapid radiation: the juxtaposed evolutionary histories of coelotine spiders support the Eocene-Oligocene orogenesis of the Tibetan Plateau. Systematic Biology 66(6): 988–1006. 10.1093/sysbio/syx042 28431105

[B24] Zhu M Wang X Zhang Z (2017) Fauna Sinica: Invertebrata Vol. 59: Arachnida: Araneae: Agelenidae and Amaurobiidae. Science Press, Beijing, 727 pp.

